# Prevalence and Associated Factors of Taking Intermittent Preventive Treatment in Pregnancy in Sierra Leone

**DOI:** 10.3390/tropicalmed4010032

**Published:** 2019-02-07

**Authors:** Amos Buh, Komlan Kota, Ghose Bishwajit, Sanni Yaya

**Affiliations:** 1 Interdisciplinary School of Health Sciences, Faculty of Health Sciences, University of Ottawa, Ottawa, ON K1N6N5, Canada; abuh020@uottawa.ca (A.B.); kkota029@uottawa.ca (K.K.); 2 School of International Development and Global Studies, University of Ottawa, Ottawa, ON K1N 6N5, Canada

**Keywords:** IPTp-SP, malaria, pregnancy, multiple indicator cluster survey, Sierra Leone

## Abstract

Malaria infection during pregnancy is a major public health problem in sub-Saharan Africa. The World Health Organization (WHO) recommends that gestational and congenital malaria can be prevented by using intermittent preventive treatment of malaria in pregnancy with sulfadoxine-pyrimethamine (IPTp-SP). IPTp-SP is a full therapeutic course of antimalarial medicine administered during pregnancy as a component of antenatal care. This study’s objective was to assess the prevalence and predictors of IPTp-SP uptake in pregnancy in Sierra Leone. This study was based on the fifth round of the Multiple Indicator Cluster Survey (MICS 5) conducted in Sierra Leone in 2016. Participants were 8526 women aged between 15–49 years. Outcome variables were uptake of IPTp-SP during the last pregnancy. Data were analysed using cross-tabulation and logistic regression methods. Results showed that the prevalence of taking IPTp-SP was 94.81% (92.40, 96.14), and that the prevalence of taking at least three doses was 93.24% (92.50, 94.81). In the multivariate logistic regression, education, parity, and antenatal care (ANC) use were significant predictors of IPTp-SP uptake. Women with higher education had lower odds of taking IPTp-SP (Odds Ratio = 0.647, 95%CI = 0.444, 0.943); having higher parity (>4) was associated with lower odds of taking IPTp-SP (OR = 0.663; 95%CI = 0.442, 0.994) and adequate ANC use increased the odds of taking IPTp-SP in both urban (OR = 1.450, 95%CI = 1.158, 3.128) and rural areas (OR = 1.903, 95%CI = 1.069, 1.966). In contrast, the positive association between ANC visits and adequate doses of taking IPTp-SP was true for rural women only (OR = 1.408, 95%CI = 1.174, 1.689). In conclusion, the use of IPTp-SP is close to being universal, with the prevalence being relatively higher in the rural areas. Based on our findings, promoting adequate antenatal care visits should be regarded as a key strategy to improve the use of IPTp-SP in Sierra Leone. Further studies could focus on exploring other predictors of IPTp-SP uptake that are not captured by MICS in Sierra Leone.

## 1. Introduction

Malaria, a parasitic infection transmitted by mosquitoes, is one of the most devastating infectious diseases, killing more than 1 million people annually. Pregnant women, children, and immunocompromised individuals have the highest morbidity and mortality [1]. Globally, 125 million women are at risk of malaria every year and in sub-Saharan Africa, the area most burdened by malaria, the disease is thought to cause as many as 10,000 cases of malaria-related deaths in pregnancy, between 75,000 and 200,000 infants deaths annually resulting from malarial infection during pregnancy, and approximately 11% (100,000) of neonatal deaths due to low birth weight resulting from *Plasmodium falciparum* infections in pregnancy [2]. 

Malaria infection during pregnancy is thus a major public health problem, with substantial risks for the mother, her foetus, and the neonate [3]. It has been reported that malaria-associated maternal illness and low birth weight is mostly the result of *Plasmodium falciparum* infection and it occurs predominantly in Africa [4]. Pregnant women are more susceptible than the general population to malaria—they are more likely to become infected, have a recurrence, develop severe complications, and to die from the disease [5].

Some of the complications suffered by pregnant women infected with malaria include miscarriages, intrauterine demise, premature delivery, low-birth-weight neonates, neonatal death, severe anaemia, hypoglycaemia, acute pulmonary oedema, foetal distress, premature labour, spontaneous abortions, and maternal death [1,6]. Besides these complications, studies have reported that some factors associated with the occurrence of malaria in pregnancy include low maternal age, low parity, low gestational age, place of residence of woman, household wealth status, maternal educational level, knowledge of malaria in pregnancy, living in congested apartments, and non-use of sulfadoxine-pyrimethamine (IPTp-SP) during pregnancy [7,8,9].

The World Health Organization (WHO) recommends the use of intermittent preventive treatment of malaria in pregnancy with IPTp-SP to prevent gestational malaria [3]. Many countries, including Sierra Leone, have adopted this policy [10]. However, although pregnant women are encouraged to take IPTp-SP [11], poor implementation of the WHO policy has been observed through low utilization rates of IPTp-SP reported by many studies [9,12,13,14].

The risk of malaria in Sierra Leone is present throughout the country, including urban areas, and the risk is present at all altitudes. The incidence of *Plasmodium falciparum* Malaria is greater than 85% [15]. Although the country has launched several initiatives, such as insecticide treated nets and IPTp-SP use among pregnant women [16,17], the rate of non-use of these cost-effective methods is high and needs further investment to ensure better efficacy of these programs. In 2015, the President’s Malaria Initiative (PMI) launched a six-year strategy, setting forth a bold and ambitious goal for malaria prevention and control in member countries. Two years later, in 2017, Sierra Leone was selected as a PMI focus country in the fiscal year (FY) 2017 [18]. In order to achieve the target of universal coverage for IPTp stated in the PMI plan, it is necessary to continuously assess the level of utilisation of IPTp-SP and its covariates, which should be used to guide policy making on the prevention and control of malaria in pregnancy. This study’s objective was therefore to assess the prevalence and predictors of IPTp-SP uptake in pregnancy in Sierra Leone. Due to lack of population-based surveys on malaria-related indicators in Sierra Leone, we used data from the latest Multiple Indicator Cluster Survey (MICS) that was conducted in Sierra Leone in 2017. The findings are expected to provide important insights for malaria control programs and promotion of IPTp in the country. 

## 2. Methods

### 2.1. Data Source

Data were collected from the sixth round of the Multiple Indicator Cluster Survey (MICS) for Sierra Leone in 2017. The survey was carried out in by Statistics Sierra Leone (Stats SL) with technical support from United Nations Children’s Fund (UNICEF) as part of the Global MICS Programme, with financial support provided by The Government of Sierra Leone, UNICEF, United Nations Population Fund (UNFPA), the World Health Organization (WHO), World Food Programme (WFP), and the European Union (EU). Field work lasted from May to August, 2017. The survey included 18,006 women aged 15–49 years, of whom 17,873 were interviewed (response rate 99.3%). However, the present study only included those experiencing a childbirth five years prior to the survey. Details of the survey have been published elsewhere (Statistics Sierra Leone. 2018. Sierra Leone Multiple Indicator Cluster Survey 2017, Survey Findings Report. Freetown, Sierra Leone: Statistics Sierra Leone). 

### 2.2. Measures

Outcome variable: The main outcome variable was adequate use of intermittent preventive therapy with sulfadoxine-pyrimethamine (IPTp-SP), which was assessed by asking the respondents whether or not they took Fansidar (sulfadoxine-pyrimethamine, SP) during their last pregnancy. As per WHO recommendations, at least three doses of SP were defined as adequate: adequate (≥3 doses) and inadequate (<3 doses).

### 2.3. Sociodemographic Variables

The following variables were considered for the analysis due to their known/theoretical association with the use of malarial treatments in the general population: 

Age groups (15–19, 20–24, 25–29, 30–34, 35–39, 40–44, 45–49); Residency (Urban, Rural); Region (North, South, West); Education (Pre-Primary/None, Primary, Lower Secondary, Upper Secondary/Higher); Ethnicity (Mende, Temne, Limba, Other); Wealth Status (Poorest, Second, Middle, Fourth, Richest); Parity (1/2, 3/4, >4); Radio Use (Not at All, Less Than Once a Week, Almost Every Day); TV Use (Not at All, Less Than Once a Week, Almost Every Day); Internet Use (Yes, No); Has Mobile (Yes, No); ANC (antenatal care) visit (Less Than four/Inadequate, >4/Adequate). The underlying assumption is that health service utilisation is influenced by women’s knowledge, self-efficacy, accessibility, and affordability of a service, which are likely to vary across sociodemographic groups and geography. We included media use variables such as TV and radio as they can improve women’s exposure to health information, thus potentially leading to service utilisation. 

All the variables were self-reported, except for the wealth index. Wealth index is calculated by assessing the possession of durable goods in the household (e.g., TV, radio, bicycle, etc.) and housing quality (e.g., type of floor, wall, roof, etc.). Each of the selected household items is assigned a factor score generated through principal component analysis (PCA), which is then summed and standardized for the households. The scores are thus obtained from a continuous scale and subsequently categorized into quintiles to rank the household as poorest/poorer/middle/richer/richest [19]. 

### 2.4. Statistical Analyses

Statistical analyses were performed with Stata version 14. Prevalence rates of adequate uptake of IPTp-SP for each explanatory variable were shown as percentages by using the survey command to account for survey weights. After that, a binary logistic regression model was used to calculate the odds ratios of the associations between adequate use of IPTp-SP and the covariates. Both bivariate and multivariate analyses were carried out to examine the crude and adjusted association. Results of regression models were presented as odds ratios along with their 95% CIs. A *p*-value of <0.05 was considered statistically significant for all analyses.

### 2.5. Ethical Approval

The study was based on the analysis of secondary data sourced from the UNICEF website: http://mics.unicef.org/. Since this website and its data are open to the public, no additional approval was necessary to reuse the data.

## 3. Results

### 3.1. Sample Characteristics and Prevalence of Uptake of IPTp-SP in Sierra Leone

Sample characteristics and the prevalence of the uptake of IPTp-SP among women in Sierra Leone are shown in Table 1. Regarding participants’ characteristics, 2112 (24.8%) of the participants were of age 25–29 years. Most of the participants (68.2%) were residents in rural areas, 3251 (38.1%) of whom were from the North region of the country and 59.1% of whom either had no education or had attained only the pre-primary level of education. Three thousand and sixty-seven (36.0%) of the participants were from the Temne ethnicity and 26.7% of them were from households in the poorest wealth quintile. Most of the participants (46.4%) had delivered twice. More than half (61.5%) of the women did not use the radio, 82.0% of them did not use the television (TV), and 96.7% of the women did not use internet services. However, 5434 (63.7%) of the participants had mobile phones and 87.5% of them made adequate ANC visits when they were pregnant.

Regarding the prevalence of IPTp-SP uptake among women, results indicate that the overall prevalence of taking IPTp-SP was 94.81% (92.40, 96.14), and that the prevalence of taking at least three doses was 93.24% (92.50, 94.81). The results also show that the percentage of taking IPTp-SP was higher in women aged 25–29 years (24.7%), residents of rural areas (59.4%), individuals in the North region (35.2%), women with primary level education (55.1%), women of Limba ethnicity (32.1%), participants from households in the poorest wealth quintile (22.0%), those who have given birth twice (47.7%), and those who respectively do not use the radio, TV, and internet (58.2%, 76.0%, and 95.7%, respectively). IPTp-SP uptake also tended to be higher in women who had no mobile phones (60.0%), as well as in women who made adequate ANC visits (88.5%).

### 3.2. Predictors of IPTp-SP Uptake Among Women in Sierra Leone

The factors associated with the uptake of IPTp-SP among women in Sierra Leone are presented in Table 2 and Table 3. In the bivariate analysis, all factor variables appeared to have an association with the uptake of IPTp-SP. These covariates included participants’ age, place of residence, region of residence, educational level, ethnicity, wealth status, use of radio, use of TV, use of internet, possession of a mobile phone, parity, and ANC visits (Table 2).

In the multivariate logistic regression, women with higher education had lower odds of taking IPTp-SP (OR = 0.647, 95%CI = 0.444, 0.943). Having access to mobile or internet did not show any significant association with IPTp-SP use. Having higher parity (>4) was associated with lower odds of taking IPTp-SP (OR = 0.663; 95%CI = 0.442, 0.994). Adequate ANC use increased the odds of taking IPTp-SP in both urban (OR = 1.450, 95%CI = 1.158, 3.128) and rural areas (OR = 1.903, 95%CI = 1.069, 1.966). In contrast, the positive association between ANC visits and adequate doses of taking IPTp-SP was true for rural women only (OR = 1.408, 95%CI = 1.174, 1.689) (Table 3). 

## 4. Discussion

In this study, we examined the prevalence and predictors of taking intermittent preventive treatment of malaria in pregnancy in Sierra Leone, using data from the sixth round of the Multiple Indicator Cluster Survey (MICS 5) conducted in Sierra Leone in 2016. The study found that the overall prevalence of IPTp-SP uptake among women in Sierra Leone was 94.81%, while the prevalence of women who took at least three doses of IPTp-SP was 93.24%. We also documented that the prevalence of IPTp-SP uptake tends to be higher in women aged 25–29, women residents in rural areas, women from the North region, women with primary level of education, women of Limba ethnicity, women from households in the poorest wealth quintile, those who have given birth twice, those who do not use the radio, TV, and internet, as well as women who had no mobile phones and who made adequate ANC visits. 

In the multivariate logistic regression analysis, results showed that the covariates that had statistically significant higher odds of prediction of IPTp-SP uptake in pregnancy included higher educational level, higher parity (>4), and making adequate ANC visits during pregnancy. Surprisingly, women with primary and higher education had lower odds of taking IPTp-SP compared with those who had no education. In general, educated women are more likely to be aware of pregnancy health-related issues and take preventive measures. For example, previous studies have shown that educated women are more likely to use reproductive health services such as antenatal and postnatal care [20,21]. Similarly, women who attend ANC have high odds of taking IPTp-SP. Our findings showed a strong positive association between ANC and taking IPTp-SP, however, the positive association between ANC and adequate doses of taking IPTp-SP was true only for rural women. We also found that women with high parity had lower odds of taking IPTp-SP. This association was also significant only among rural women; a possible reason for this might be the lower socioeconomic condition associated with higher parity in many African countries. 

The high prevalence of IPTp-SP uptake reported in this study falls in line with the findings of a similar study in Ghana that reported a high IPTp-SP uptake level of 98.5% [22]. Nevertheless, the estimated prevalence of IPTp-SP uptake reported in this study is higher than the prevalence range of 29.5–70.2% reported in other studies [9,12,13,14]. Also, the prevalence of taking at least three doses of IPTp-SP during pregnancy reported in this study is similar to that reported in a study conducted in Tanzania [23], although it differs greatly with the findings of other studies that reported lower prevalence of adequate IPTp-SP uptake [9,12,14,24].

With regards to the predictors of IPTp-SP uptake, some studies have reported that the statistically significant factors associated with IPTp-SP uptake include institutional delivery, attendance of ANC, awareness of IPTp-SP, having placental malaria, and having higher educational level [12,24,25]. However, our study reveals that higher educational level of women, higher parity, and adequate ANC use by women were significant factors associated with adequate IPTp-SP uptake. This is similar to the findings of studies that have found educational level [12,24,26,27], ANC attendance [9,24], and parity [28] as significant factors of adequate IPTp-SP uptake. However, the findings are dissimilar to those of a study that found only placental parasitaemia as an associated factor to uptake of IPTp-SP [29]. In Sierra Leone, the health system is well organized, and it uses the PMI strategic plan for FY 2017 [18] to prevent and control malaria in women who are pregnant through the distribution of sulfadoxine pyrimethamine to pregnant women at their ANC visits. We perceive this to be the main force behind the high prevalence of IPTp-SP uptake in this country. 

## 5. Strengths and Limitations

Our findings might be limited considering the secondary nature of the data we used, which allowed us little control over the variables to include in our analysis, and the fact that we considered only age, place of residence, region, education, ethnicity, wealth status, use of radio, TV, and internet, as well as possession of mobile phone, and parity visits as potential predictors of IPTp-SP uptake. Other predictors of IPTp-SP uptake not captured by the MICS might have been missed. For example, sociocultural factors such as trust or misconception about drugs/vaccines are known predictors of drug/vaccine hesitancy among women [30,31]. We were also unable to assess whether there were any geographic barriers to accessing the services in the study population, which might have influenced the association. However, we worked within the confines of our objectives, and other predictors of IPTp-SP uptake could be explored in another study.

## 6. Conclusions

The present analysis concludes that the use of IPTp-SP is close to being universal in Sierra Leone, with the prevalence being relatively higher in the rural areas. Based on our findings, promoting adequate antenatal care visits by ensuring the availability of IPTp drugs should be regarded as an important strategy to improve the use of IPTp-SP in Sierra Leone. Further studies could focus on exploring other predictors of IPTp-SP uptake that are not captured by MICS in Sierra Leone.

## Figures and Tables

**Table 1:** Prevalence of taking sulfadoxine-pyrimethamine (IPTp-SP) in Sierra Leone (*n* = 8526).

-	-	Took IPTp-SP 94.81% [92.40, 96.14]		-	At Least 3 Doses of IPTp-SP 93.24% [92.50, 94.81]		-
**Age Groups**	***N*, %**	**No**	**Yes**	***p*-Value**	**No**	**Yes**	***p*-Value**
15–19	799, 9.4	11.5 [8.4, 15.5]	8.8 [8.1, 9.5]	0.015	8.4 [7.7, 9.3]	9.7 [8.4, 11.2]	0.009
20–24	2019, 23.7	27.2 [22.4, 32.6]	23.9 [22.9, 25.0]		23.8 [22.5, 25.0]	24.5 [22.5, 26.6]	
25–29	2112, 24.8	24.1 [19.8, 29.1]	24.7 [23.7, 25.8]		25.7 [24.4, 27.0]	22.2 [20.4, 24.3]	
30–34	1612, 18.9	17.0 [13.5, 21.4]	19.5 [18.5, 20.5]		19.2 [18.1, 20.4]	20.1 [18.3, 22.0]	
35–39	1261, 14.8	12.1 [8.9, 16.3]	14.9 [14.1, 15.9]		15.1 [14.1, 16.2]	14.5 [12.9, 16.3]	
40–44	522, 6.1	6.7 [4.5, 9.9]	5.7 [5.2, 6.3]		5.6 [5.0, 6.3]	6.1 [5.1, 7.2]	
45–49	201, 2.4	1.3 [0.6, 2.6]	2.4 [2.0, 2.8]		2.2 [1.8, 2.7]	2.9 [2.2, 3.8]	
**Residency**							
Urban	2708, 31.8	46.0 [40.2, 52.0]	40.6 [39.1, 42.0]	0.003	41.7 [40.0, 43.4]	37.4 [34.9, 40.0]	0.061
Rural	5818, 68.2	54.0 [48.0, 59.8]	59.4 [58.0, 60.9]		58.3 [56.6, 60.0]	62.6 [60.0, 65.1]	
**Region**							
East	1921, 22.5	20.2 [16.3, 24.7]	23.5 [22.4, 24.7]	<0.001	24.4 [23.1, 25.7]	21.2 [19.3, 23.2]	<0.001
North	3251, 38.1	37.6 [32.2, 43.4]	35.2 [33.9, 36.5]		34.3 [32.8, 35.8]	37.7 [35.4, 40.0]	
South	2085, 24.5	13.8 [10.9, 17.3]	19.6 [18.6, 20.6]		18.1 [17.0, 19.2]	23.6 [21.7, 25.7]	
West	1269, 14.9	28.4 [22.9, 34.6]	21.7 [20.4, 23.1]		23.2 [21.7, 24.9]	17.5 [15.4, 19.8]	
**Education**							
Pre-Primary/None	5041, 59.1	46.3 [40.7, 51.9]	55.1 [53.8, 56.4]	<0.001	55.0 [53.5, 56.5]	55.3 [52.9, 57.6]	<0.001
Primary	1168, 13.7	19.3 [15.0, 24.5]	13.6 [12.7, 14.5]		13.4 [12.4, 14.4]	14.1 [12.6, 15.8]	
Lower Secondary	1314, 15.4	16.5 [12.2, 22.0]	16.4 [15.4, 17.3]		16.4 [15.3, 17.6]	16.2 [14.5, 18.0]	
Upper Secondary/Higher	1003, 11.8	17.9 [13.5, 23.2]	15.0 [14.0, 16.1]		15.2 [14.0, 16.5]	14.4 [12.6, 16.5]	
**Ethnicity**							
Mende	2270, 26.6	26.7 [22.0, 32.0]	26.3 [25.1, 27.5]	<0.001	26.1 [24.7, 27.5]	26.9 [24.8, 29.2]	<0.001
Temne	3067, 36.0	24.7 [20.1, 29.9]	33.7 [32.4, 35.0]		33.1 [31.6, 34.6]	35.4 [33.1, 37.8]	
Limba	2562, 30.0	40.0 [34.4, 45.9]	32.1 [30.8, 33.5]		32.5 [31.0, 34.1]	31.1 [28.9, 33.4]	
Other	627, 7.4	8.6 [5.9, 12.4]	7.8 [7.1, 8.6]		8.3 [7.5, 9.3]	6.5 [5.4, 7.9]	
**Wealth Status**							
Poorest	2280, 26.7	22.8 [18.9, 27.3]	22.0 [21.0, 23.0]	<0.001	21.8 [20.7, 23.0]	22.4 [20.6, 24.3]	<0.001
Second	2047, 24.0	17.9 [14.4, 22.1]	21.3 [20.3, 22.3]		21.0 [19.8, 22.2]	22.2 [20.3, 24.1]	
Middle	1890, 22.2	20.4 [15.7, 25.9]	20.4 [19.4, 21.5]		19.9 [18.7, 21.2]	21.9 [20.0, 23.9]	
Fourth	1302, 15.3	17.0 [13.0, 21.9]	19.2 [18.0, 20.4]		20.2 [18.8, 21.7]	16.4 [14.5, 18.5]	
Richest	1007, 11.8	21.9 [16.9, 27.8]	17.1 [16.0, 18.4]		17.1 [15.8, 18.6]	17.2 [15.1, 19.5]	
**Parity**							
1/2	3954, 46.4	50.1 [44.4, 55.8]	47.7 [46.5, 49.0]	<0.001	47.5 [46.0, 49.0]	48.2 [45.8, 50.6]	<0.001
3/4	2679, 31.4	27.9 [23.4, 32.8]	30.9 [29.7, 32.0]		31.6 [30.2, 33.0]	28.9 [26.9, 31.1]	
>4	1893, 22.2	22.0 [17.8, 26.8]	21.4 [20.4, 22.4]		20.9 [19.7, 22.1]	22.9 [21.0, 24.8]	
**Radio Use**							
Not at All	5244, 61.5	58.9 [53.0, 64.6]	58.2 [56.9, 59.5]	0.023	58.7 [57.1, 60.2]	57.0 [54.5, 59.4]	0.035
Less Than Once a Week	2327, 27.3	30.5 [25.0, 36.5]	29.1 [27.9, 30.3]		29.7 [28.3, 31.1]	27.5 [25.3, 29.7]	
Almost Every Day	955, 11.2	10.6 [7.8, 14.2]	12.7 [11.8, 13.6]		11.6 [10.6, 12.7]	15.6 [13.8, 17.5]	
**TV Use**							
Not at All	6995, 82.0	70.0 [64.1, 75.3]	76.0 [74.7, 77.2]	<0.001	76.0 [74.5, 77.5]	75.9 [73.5, 78.1]	<0.001
Less Than Once a Week	998, 11.7	18.1 [13.7, 23.5]	14.7 [13.7, 15.8]		14.8 [13.6, 16.1]	14.5 [12.7, 16.5]	
Almost Every Day	533, 6.3	11.9 [8.3, 16.9]	9.3 [8.4, 10.2]		9.2 [8.1, 10.3]	9.6 [8.1, 11.4]	
**Internet Use**							
Yes	278, 3.3	3.4 [1.8, 6.3]	4.3 [3.8, 5.0]	<0.001	4.7 [4.0, 5.5]	3.3 [2.4, 4.5]	<0.001
No	8248, 96.7	96.6 [93.7, 98.2]	95.7 [95.0, 96.2]		95.3 [94.5, 96.0]	96.7 [95.5, 97.6]	
**Has Mobile**							
Yes	3092, 36.3	40.1 [34.5, 45.9]	40.0 [38.7, 41.3]		40.8 [39.2, 42.3]	37.9 [35.5, 40.3]	<0.001
No	5434, 63.7	59.9 [54.1, 65.5]	60.0 [58.7, 61.3]		59.2 [57.7, 60.8]	62.1 [59.7, 64.5]	
**ANC Visit**							
Inadequate	1070, 12.5	17.4 [13.7, 21.8]	11.5 [10.8, 12.4]	<0.001	12.2 [11.3, 13.2]	9.7 [8.4, 11.1]	<0.001
Adequate	7456, 87.5	82.6 [78.2, 86.3]	88.5 [87.6, 89.2]		87.8 [86.8, 88.7]	90.3 [88.9, 91.6]	

N.B. *p*-values are from chi-squared tests.

**Table 2:** Predictors of taking IPTp-SP among urban and rural women in Sierra Leone (bivariate analysis).

-	Total	Urban	Rural
**Age groups (15–19)**	1	1	1
20–24	1.209	1.435	1.087
	[0.839, 1.742]	[0.793, 2.595]	[0.679, 1.740]
25–29	1.318	1.585	1.182
	[0.890, 1.951]	[0.829, 3.032]	[0.717, 1.947]
30–34	1.436	1.709	1.311
	[0.923, 2.236]	[0.819, 3.566]	[0.746, 2.305]
35–39	1.646^*^	2.255	1.407
	[1.016, 2.667]	[0.974, 5.223]	[0.771, 2.568]
40–44	1.327	2.096	1.092
	[0.760, 2.316]	[0.730, 6.024]	[0.557, 2.143]
45–49	2.075	4.714	1.620
	[0.909, 4.739]	[0.558, 39.81]	[0.642, 4.090]
**Residency (Urban)**	1	-	-
Rural	1.371	NA	NA
	[0.974, 1.932]	-	-
**Regions (East)**	1	1	1
North	1.335	0.595	1.729 **
	[0.970, 1.837]	[0.306, 1.158]	[1.202, 2.486]
South	1.027	0.575	1.118
	[0.749, 1.409]	[0.297, 1.115]	[0.774, 1.613]
West	1.035	0.502 *	1.439
	[0.692, 1.547]	[0.272, 0.928]	[0.485, 4.267]
**Education (None)**	1	1	1
Primary	0.709 *	0.507 **	0.854
	[0.536, 0.938]	[0.314, 0.820]	[0.597, 1.221]
Secondary	0.920	1.061	0.828
	[0.667, 1.268]	[0.621, 1.815]	[0.551, 1.242]
Higher	0.647 *	0.622	0.769
	[0.444, 0.943]	[0.375, 1.033]	[0.387, 1.528]
**Ethnicity (Other)**	1	1	1
Mende	1.661 **	0.759	2.348 ***
	[1.217, 2.268]	[0.438, 1.317]	[1.616, 3.413]
Temne	0.789	0.613 *	0.881
	[0.607, 1.026]	[0.392, 0.958]	[0.632, 1.229]
Limba	0.862	0.688	0.932
	[0.581, 1.280]	[0.361, 1.312]	[0.560, 1.551]
**Wealth (Poorest)**	1	1	1
Poorer	1.220	0.511	1.244
	[0.922,1.614]	[0.0941, 2.770]	[0.933, 1.659]
Middle	1.194	0.512	1.335
	[0.882, 1.615]	[0.114, 2.307]	[0.952, 1.872]
Higher	1.846 **	1.166	0.799
	[1.168, 2.917]	[0.257, 5.288]	[0.413, 1.546]
Highest	1.623	0.951	1.036
	[0.931, 2.830]	[0.202, 4.481]	[0.281, 3.823]
**Radio (Never)**	1	1	1
Few days a week	1.142	1.022	1.193
	[0.892, 1.461]	[0.688, 1.519]	[0.859, 1.656]
Almost everyday	1.259	1.125	1.260
	[0.879, 1.804]	[0.661, 1.913]	[0.752, 2.111]
**TV (Never)**	1	1	1
Few days a week	0.761	0.618 *	1.294
	[0.541, 1.070]	[0.401, 0.952]	[0.657, 2.548]
Almost every day	0.824	0.761	0.957
	[0.490, 1.386]	[0.423, 1.367]	[0.200, 4.582]
**Internet Use (No)**	1	1	1
Yes	0.668	0.548	1.379
	[0.359, 1.244]	[0.253, 1.185]	[0.491, 3.872]
**Has Mobile (No)**	1	1	1
Yes	1.036	1.192	0.962
	[0.815, 1.317]	[0.816, 1.743]	[0.697,1.327]
**Parity (1/2)**	1	1	1
3/4	0.828	0.753	0.897
	[0.630, 1.088]	[0.472, 1.202]	[0.639, 1.258]
>4	0.620 **	0.613	0.663 *
	[0.441, 0.872]	[0.319, 1.178]	[0.442, 0.994]
**ANC Visit (Inadequate)**	1	1	1
Adequate	1.556 ***	1.903 *	1.450 *
	[1.202, 2.014]	[1.158, 3.128]	[1.069, 1.966]

N.B. Odds ratios; 95% confidence intervals in brackets. * *p* < 0.05, ** *p* < 0.01, *** *p* < 0.001.

**Table 3:** Predictors of taking adequate doses of IPTp-SP among urban and rural women in Sierra Leone (multivariate analysis).

	Total	Urban	Rural
**Age groups (15–19)**	1	1	1
20–24	0.958	1.124	0.905
	[0.790, 1.162]	[0.787, 1.605]	[0.717, 1.142]
25–29	0.908	0.947	0.907
	[0.740, 1.115]	[0.647, 1.386]	[0.709, 1.159]
30–34	1.097	1.202	1.072
	[0.875, 1.376]	[0.790, 1.830]	[0.817, 1.406]
35–39	0.909	0.937	0.905
	[0.712, 1.161]	[0.588, 1.493]	[0.677, 1.210]
40–44	1.144	1.168	1.150
	[0.855, 1.530]	[0.655, 2.082]	[0.819, 1.617]
45–49	1.192	1.607	1.068
	[0.819, 1.733]	[0.730, 3.538]	[0.695, 1.642]
**Residency (Urban)**	1	-	-
Rural	1.007	NA	NA
	[0.842,1.205]	-	-
**Regions (East)**	1	1	1
North	1.497 ***	1.493 *	1.479 ***
	[1.260, 1.779]	[1.076, 2.070]	[1.202, 1.820]
South	1.167 *	1.477 *	1.132
	[1.001, 1.361]	[1.072,2.034]	[0.947,1.354]
West	0.781 *	0.921	0.536
	[0.626, 0.975]	[0.685, 1.238]	[0.271, 1.060]
**Education (None)**	1	1	1
Primary	1.099	0.886	1.182
	[0.943, 1.281]	[0.652, 1.206]	[0.989, 1.412]
Secondary	1.028	1.037	0.999
	[0.876, 1.207]	[0.792, 1.356]	[0.816, 1.224]
Higher	0.978	0.850	1.052
	[0.797, 1.200]	[0.642, 1.126]	[0.743, 1.490]
**Ethnicity (Other)**	1	1	1
Mende	1.028	1.232	0.951
	[0.875, 1.208]	[0.939, 1.616]	[0.777, 1.165]
Temne	0.899	0.862	0.912
	[0.782, 1.034]	[0.676, 1.100]	[0.768, 1.083]
Limba	0.684 ***	1.080	0.541 ***
	[0.546, 0.856]	[0.752, 1.551]	[0.405, 0.722]
**Wealth (Poorest)**	1	1	1
Poorer	1.031	0.536	1.036
	[0.896, 1.186]	[0.224, 1.280]	[0.897, 1.195]
Middle	1.123	0.730	1.087
	[0.966, 1.306]	[0.364, 1.464]	[0.925, 1.279]
Higher	1.107	0.612	1.247
	[0.879, 1.395]	[0.307, 1.219]	[0.855, 1.819]
Highest	1.199	0.636	2.328 **
	[0.897, 1.604]	[0.309, 1.308]	[1.316, 4.119]
**Radio (Never)**	1	1	1
Few days a week	1.048	1.231	0.978
	[0.925, 1.188]	[0.986, 1.536]	[0.837, 1.142]
Almost every day	1.453 ***	1.393 *	1.492 ***
	[1.226, 1.722]	[1.053, 1.842]	[1.196, 1.863]
**TV (Never)**	1	1	1
Few days a week	1.180	0.985	1.460 **
	[0.981, 1.419]	[0.770, 1.261]	[1.097, 1.942]
Almost every day	1.329 *	1.296	1.065
	[1.008, 1.752]	[0.947, 1.772]	[0.498, 2.280]
**Internet Use (No)**	1	1	1
Yes	1.299	1.149	1.756
	[0.957, 1.764]	[0.802, 1.648]	[0.936, 3.294]
**Has Mobile (No)**	1	1	1
Yes	1.208 **	1.060	1.306 ***
	[1.068, 1.368]	[0.860, 1.308]	[1.115, 1.530]
**Parity (1/2)**	1	1	1
3/4	0.922	0.901	0.936
	[0.804, 1.057]	[0.698, 1.162]	[0.795, 1.102]
>4	1.065	1.069	1.066
	[0.898, 1.263]	[0.753, 1.517]	[0.875, 1.299]
**ANC Visit (Inadequate)**	1	1	1
Adequate	1.417 ***	1.454	1.408 ***
	[1.204, 1.668]	[0.999, 2.116]	[1.174, 1.689]

N.B. Odds ratios; 95% confidence intervals in brackets; * *p* < 0.05, ** *p* < 0.01, *** *p* < 0.001.

## Data Availability

Data for this study were sourced from the UNICEF website: http://mics.unicef.org/.

## Abbreviations

ANC	Antenatal care
CI	Confidence interval
IPTp-SP	Intermittent preventive treatment of malaria during pregnancy with sulfadoxine-pyrimethamine
OR	Odds ratio
MICS	Multiple indicator cluster survey
N	Frequency
SP	Sulfadoxine pyrimethamine
TV	Television
WHO	World Health Organization
